# Literature review of stroke assessment for upper-extremity physical function via EEG, EMG, kinematic, and kinetic measurements and their reliability

**DOI:** 10.1186/s12984-023-01142-7

**Published:** 2023-02-15

**Authors:** Rene M. Maura, Sebastian Rueda Parra, Richard E. Stevens, Douglas L. Weeks, Eric T. Wolbrecht, Joel C. Perry

**Affiliations:** 1 Mechanical Engineering Department, University of Idaho, Moscow, ID USA; 2 Engineering and Physics Department, Whitworth University, Spokane, WA USA; 3 College of Medicine, Washington State University, Spokane, WA USA; 4 Electrical Engineering Department, University of Idaho, ID Moscow, USA

**Keywords:** Stroke, Reliability, Robot-assisted therapy, Exoskeleton, Neurological assessment, Biomechanical assessment, Rehabilitation, Motor function, Electroencephalography, Multimodal

## Abstract

**Background:**

Significant clinician training is required to mitigate the subjective nature and achieve useful reliability between measurement occasions and therapists. Previous research supports that robotic instruments can improve quantitative biomechanical assessments of the upper limb, offering reliable and more sensitive measures. Furthermore, combining kinematic and kinetic measurements with electrophysiological measurements offers new insights to unlock targeted impairment-specific therapy. This review presents common methods for analyzing biomechanical and neuromuscular data by describing their validity and reporting their reliability measures.

**Methods:**

This paper reviews literature (2000–2021) on sensor-based measures and metrics for upper-limb biomechanical and electrophysiological (neurological) assessment, which have been shown to correlate with clinical test outcomes for motor assessment. The search terms targeted robotic and passive devices developed for movement therapy. Journal and conference papers on stroke assessment metrics were selected using PRISMA guidelines. Intra-class correlation values of some of the metrics are recorded, along with model, type of agreement, and confidence intervals, when reported.

**Results:**

A total of 60 articles are identified. The sensor-based metrics assess various aspects of movement performance, such as smoothness, spasticity, efficiency, planning, efficacy, accuracy, coordination, range of motion, and strength. Additional metrics assess abnormal activation patterns of cortical activity and interconnections between brain regions and muscle groups; aiming to characterize differences between the population who had a stroke and the healthy population.

**Conclusion:**

Range of motion, mean speed, mean distance, normal path length, spectral arc length, number of peaks, and task time metrics have all demonstrated good to excellent reliability, as well as provide a finer resolution compared to discrete clinical assessment tests. EEG power features for multiple frequency bands of interest, specifically the bands relating to slow and fast frequencies comparing affected and non-affected hemispheres, demonstrate good to excellent reliability for populations at various stages of stroke recovery. Further investigation is needed to evaluate the metrics missing reliability information. In the few studies combining biomechanical measures with neuroelectric signals, the multi-domain approaches demonstrated agreement with clinical assessments and provide further information during the relearning phase. Combining the reliable sensor-based metrics in the clinical assessment process will provide a more objective approach, relying less on therapist expertise. This paper suggests future work on analyzing the reliability of metrics to prevent biasedness and selecting the appropriate analysis.

## Background

Stroke is one of the leading causes of death and disability in developed countries. In the United States, a stroke occurs every 40 s, ranking stroke as the fifth leading cause of death and the first leading cause of disability in the country [1]. The high prevalence of stroke, coupled with increasing stroke survival rates, puts a growing strain on already limited healthcare resources; the cost of therapy is elevated [2] and restricted mostly to a clinical setting [3], leading to 50% of survivors that reach the chronic stage experiencing severe motor disability for upper extremities [4]. This highlights the need for refined (improved) assessment which can help pair person-specific impairment with appropriately targeted therapeutic strategies.

Rehabilitation typically starts with a battery of standardized tests to assess impairment and function. This initial evaluation serves as a baseline of movement capabilities and usually includes assessment of function during activities of daily living (ADL). Because these clinical assessments rely on trained therapists as raters, the scoring scale is designed to be discrete and, in some cases, bounded. While this improves the reliability of the metric [5] (i.e., raters more likely to agree), it also reduces the sensitivity of the scale. Furthermore, those assessment scales that are bounded, such as the Fugl-Meyer Assessment (FMA) [6], Ashworth or Modified Ashworth (MA) Scale [7], and Barthel Index [8], suffer from floor/ceiling effects where the limits of the scales become insensitive to the extremes of impairment and function. It is therefore important to develop new clinical assessment methods that are objective, quantifiable, reliable, and sensitive to change over the full range of function and impairment.

**Fig. 1:**
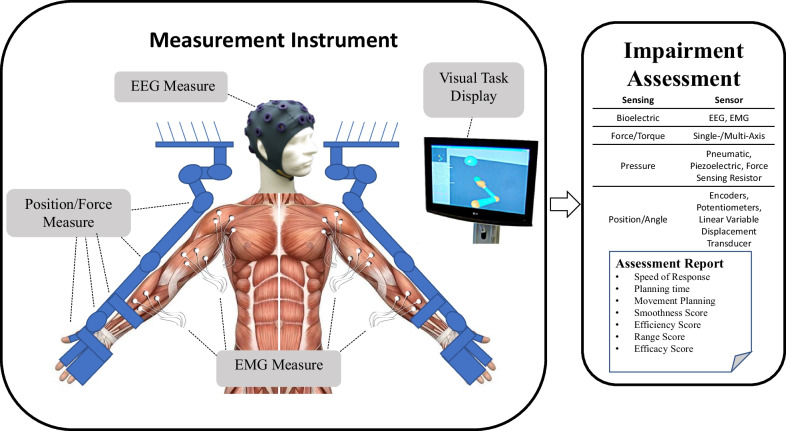
Example of instrument for upper extremities bilateral biomechanical and neuromuscular assessment. From this data, a wide variety of measures and metrics for assessment of upper-extremity impairment and function may be reported

Over the last several decades, robotic devices have been designed and studied for administering post-stroke movement therapy. These devices have begun being adopted into clinical rehabilitation practice. More recently, researchers have proposed and studied the use of robotic devices to assess stroke-related impairments as an approach to overcome the limitations of existing clinical measures previously discussed [9–12]. Robots may be equipped with sensitive measurement devices that can be used to rate the person’s performance in a predefined task. These devices can include measuring kinematic (position/velocity), kinetic (force/torque), and/or neuromuscular (electromyography/electroencephalography) output from the subject during the task. Common sensor-based robotic metrics for post-stroke assessment included speed of response, planning time, movement planning, smoothness, efficiency, range, and efficacy [13, 14]. Figure 1 demonstrates an example method for comprehensive assessment of a person who has suffered a stroke with data acquired during robotically administered tests. Furthermore, there is potential for new and more comprehensive knowledge to be gained from a wider array of assessment methods and metrics that combine the benefits of biomechanical (e.g., kinematic and kinetic) and neurological (e.g., electromyographic and electroencephalographic) measures [15–22].

### Biomechanical assessment

Many classical methods of assessing impairment or function involve manual and/or instrumented quantification of performance through measures of motion (i.e., kinematic) and force (i.e., kinetic) capabilities. These classical methods rely on the training of the therapist to evaluate the capabilities of the person through keen observation (e.g., FMA [6] and MA [7]). The quality of kinematic and kinetic measures can be improved with the use of electronic-based measurements [23]. Robotic devices equipped with electronic sensors have the potential to improve the objectivity, sensitivity, and reliability of the assessment process by providing a means for more quantitative, precise, and accurate information [9–12, 24–28]. Usually, the electronic sensors on a rehabilitation robotic device are used for control purposes [29–31]. Robotics can also measure movement outputs, such as force or joint velocities, which the clinician may not be able to otherwise measure as accurately (or simultaneously) using existing clinical assessment methods [23]. With accurate and repeatable measurement of forces and joint velocities, sensor-based assessments have the potential to assess the person’s movement in an objective and quantifiable way. This article reviews validity and reliability of biomechanical metrics in relationship to assessment of motor function for upper extremities.

### Electrophysiological features for assessment

Neural signals that originate from the body can be measured using non-invasive methods. Among others, electroencephalograms (EEG) measure cortical electrical activity, and electromyograms (EMG) measure muscle electrical activity. The relative low cost, as well as the noninvasive nature of these technologies make them suitable for studying changes in cortical or muscle activation caused by conditions or injuries of the brain, such as the ones elicited by stroke lesions [32].

Initially, EMG/EEG were used strictly as clinical diagnostic tools [33, 34]. Recent improvements in signal acquisition hardware and computational processing methods have increased their use as viable instruments for understanding and treating neuromuscular diseases and neural conditions [32]. Features extracted from these signals are being researched to assess their relationship to motor and cognitive deficits [35–42] and delayed ischemia [34, 43], as well as to identify different uses of the signals that could aid rehabilitation [44]. Applications of these features in the context of stroke include: (1) commanding robotic prostheses [45, 46], exoskeletons [21, 47, 48], and brain-machine interfaces [44, 49–51]; and (2) bedside monitoring for sub-acute patients and thrombolytic therapy [52–54]. Here we review the validity and reliability of metrics derived from electrophysiological signals in relationship to stroke motor assessment for upper extremity.

### Reliability of metrics

Robotic or sensor-based assessment tools have not gained widespread clinical acceptance for stroke assessment. Numerous barriers to their clinical adoption remain, including demonstrating their reliability and providing sufficient validation of robotic metrics with respect to currently accepted assessment techniques [55]. In the assessment of motor function with sensor-based systems, several literature reviews reveal a wide spectrum of sensor-based metrics to use for stroke rehabilitation and demonstrate their validity [13, 42, 56–59, 63, 64]. However, in addition to demonstrating validity, new clinical assessments must also demonstrate good or excellent reliability in order to support their adoption in the clinical field. This is achieved by: (1) comparing multiple measurements on the same subject (test–retest reliability), and (2) checking agreement between multiple raters of the same subject (inter-rater reliability). Reliability quantifies an assessment’s ability to deliver scores that are free from measurement error [65]. Previous literature reviews have presented limited, if any, information on the reliability of the biomechanical robotic metrics. Murphy and Häger [66], Wang et al. [56], and Shishov et al. [67] reviewed reliability, but omitted some important aspects of intra-class correlation methods used in the study (e.g., the model type and/or the confidence interval), which are required when analyzing intra-class correlation methods for reliability [68]. If the reliability is not properly analyzed and reported, the study runs the risk of having a biased result. Murphy and Häger [66] also found a lack of studies determining the reliability of metrics in 2015. Since electronic-based assessments require the use of a therapist or an operator to administer the test, an inter-observer reliability test should be investigated to observe the effect of the test administrators on the assessment process. Therefore, both test–retest and inter-observer reliability in biomechanical and electrophysiological metrics are reviewed to provide updated information on the current findings of the metrics’ reliability.

### Integrated metrics

Over the past 50 years, numerous examples of integrated metrics have provided valuable insight into the inner workings of human arm function. In the 1970s EMG was combined with kinematic data in patients with spasticity to understand muscle patterns during ballistic arm reach movements [69], the affects of pharmacological intervention on spastic stretch reflexes during passive vs. voluntary movement [70], and in the 1990s EMG was combined with kinetic data to understand the effects of abnormal synergy patterns on reach workspace when lifting the arm against gravity [71]. This work dispelled long-standing theories of muscular weakness and spasticity alone being the major contributors to arm impairment. More recently, quantified aspects of processed EEG and EMG signals are being combined with kinematic data to investigate the compensatory role, and relation to shoulder-related abnormal muscle synergies of the contralesional secondary sensorimotor cortex, in a group of chronic stroke survivors [72]. These and other works demonstrate convincingly the value of combined metrics and the insights they can uncover that isolated metrics cannot discover alone.

To provide further information on the stroke severity and the relearning process during stroke therapy, researchers are investigating a multi-modal approach using biomechanical and neuromuscular features [15, 16, 18, 19, 21, 22]. Combining both neuromuscular and biomechanical metrics will provide a comprehensive assessment of the person’s movement starting from motor planning to the end of motor execution. Neuromuscular output provides valuable information on the feedforward control and the movement planning phase [22]. However, neuromuscular signals provides little information on the movement quality that is often investigated with movement function tests or biomechanical output [21]. Also, using neuromuscular data will provide information to therapist on the neurological status and nervous system reorganization of the person that biomechanical information cannot provide [73]. The additional information can assist in developing more personalized care for the person with stroke, as well as offer considerable information on the changes that occur at the physiological level.

### Paper overview

This paper reviews published sensor-based methods, for biomechanical and neuromuscular assessment of impairment and function after neurological damage, and how the metrics resulting from the assessments, both alone and in combination, may be able to provide further information on the recovery process. Specifically, methods and metrics utilizing digitized kinematic, kinetic, EEG, and EMG data were considered. The “Methods” section explains how the literature review was performed. In “Measures and methods based on biomechanical performance” section, prevailing robotic assessment metrics are identified and categorized including smoothness, resistance, efficiency, accuracy, efficacy, planning, range-of-motion, strength, inter-joint coordination, and intra-joint coordination. In “Measures and methods based on neural activity using EEG/EMG” section, EEG- and EMG-derived measures are discussed by the primary category of analysis performed to obtain them, including frequency power and coherence analyses. The relationship of each method and metric to stroke impairment and/or function is also discussed. Section “Reliability of measures” discusses the reliability of sensor-based metrics and some of the complications in demonstrating the effectiveness of the metrics. Section “Integrated metrics” reviews previous studies on combining biomechanical and neuromuscular data to provide further information on the changes occurring during assessment and training. Finally, Section “Discussions and conclusions” concludes the paper with a discussion on the advantages of combining multi-domain data, which of the metrics from the earlier sections should be considered in future robotic applications, as well as the ones that still require more investigation for either validity and/or reliability.

## Methods

**Fig. 2:**
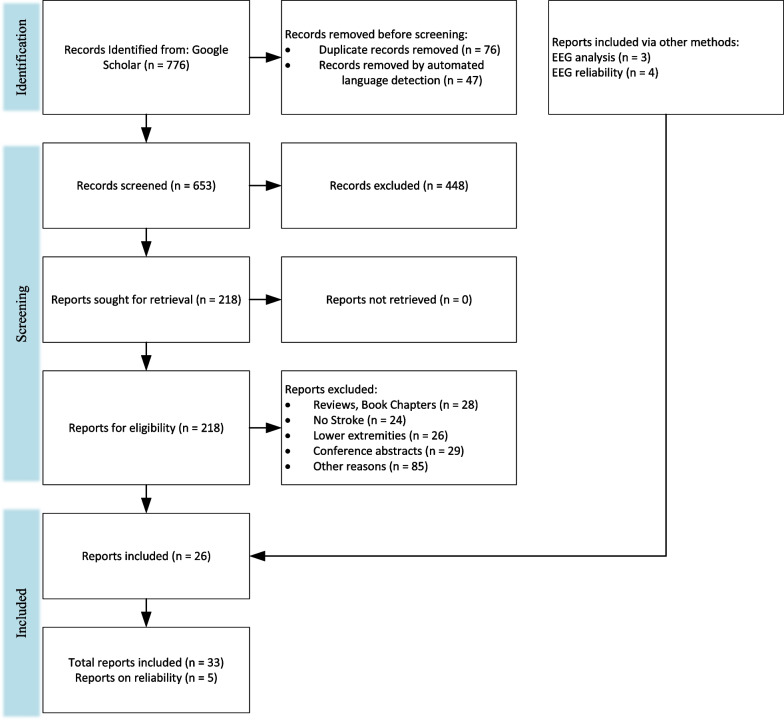
PRISMA flowchart on the selection for electrophysiological papers

**Fig. 3:**
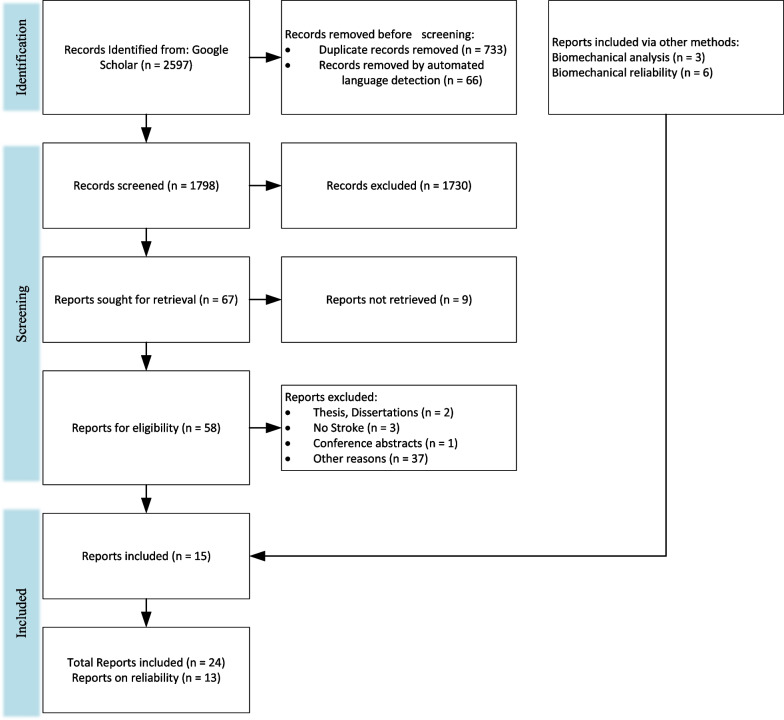
PRISMA flow chart for the selection for biomechanical papers

A literature review was performed following PRISMA guidelines [74] on biomechanical and neuromuscular assessment in upper-limb stroke rehabilitation. The review was composed of two independent searches on (1) biomechanical robotic devices, and (2) electrophysiological digital signal processing. Figures 2 and 3 show the selection process of the electrophysiological and biomechanical papers, respectively. Each of these searches applied the following steps: In step 1, each researcher searched in Google Scholar for papers between 2000 and 2021 (see Table 1 for search terms and prompts). In step 2, resulting titles and abstracts were screened to remove duplicates, articles in other languages, and articles not related to the literature review. In step 3, researchers read the full texts of articles screened in step 2, papers qualifying for inclusion using the Literature Review Criteria in Table 1 were selected. Finally, in step 4, selected articles from independent review process were read by the other researcher. Uncertainties in determining if a paper should be included/excluded were discussed with the whole research group. Twenty-four papers focus on biomechanical measures (kinematic and kinetic), thirty-three focus on electrophysiological measures (EEG/EMG), and six papers on multimodal approaches combining biomechanical and neuromuscular measures to assess stroke. Three of the six multimodal papers are also reported in the biomechanical section and 3 papers were hand-picked. A total of 60 papers are reviewed and reported.

**Table 1:** Literature review criteria

	Kinematic and kinetic measures	Neuromuscular measures (EEG/EMG)
Keywords	Stroke OR Stroke Assessment & Upper Limb Exoskeleton OR Upper Limb Robot OR Sensor Based & Relability OR Test Retest Reliability OR Inter Rater Reliability -gait	Stroke & EEG & EMG & assessment & motor function & upper extremity & corticomuscular coherence & CMC
	Stroke Rehabilitation & Electroencephalography & Kinematic & reliability & robotics -gait	Stroke & EEG & EMG & assessment & motor function & QEEG & poststroke) OR (stroke + EEG OR EMG & assessment & "motor function" & "upper extremity" & QEEG & poststroke
	Stroke Rehabilitation & Upper Limb Exoskeleton OR Upper Limb Robot OR Sensor Based AND Reliability OR Test Retest Reliability OR Inter Rater Reliability -gait	Stroke & EEG & EMG & (Multimodal or Multidomain) & Robotic Assessment & motor function & (reliability or ICC)
	Stroke Rehabilitation OR Stroke Assessment & Upper Limb Exoskeleton OR Upper Limb Robot OR Sensor Based & Reliability OR Test Retest Reliability OR Inter Rater Reliability -gait -TMS	("upper extremity" OR "upper-limb") & ("motor score" OR "motor function" OR "motor recovery") & ("connectivity measures" & "functional connectivity" OR "cortical connectivity" OR coherent OR coupling OR network) & biomarkers & (stroke OR "acute stroke “ OR “chronic stroke”)
Inclusion	Has been shown to be valid by evidence of relationship to standard assessments. The methods section was screened to include groups with various levels of impairment due to stroke, and during different stroke stages (acute, subacute and chronic). Records included for reliability must perform Intra-Class Correlation (ICC) for multiple sessions (test–retest) and/or raters (inter-rater)	
Exclusion	Studies prior to 2000 Literature reviews Lower limb, trunk displacement, and finger studies Articles not written in English Functional Electrical Stimulation	
Database	Google Scholar, JNER, PubMed, IEEE Xplore, SAGE, Frontiers for Neuroscience	

## Measures and methods based on biomechanical performance

This review presents common robotic metrics which have been previously used to assess impairment and function after stroke. Twenty-five biomechanical papers are reviewed, which used both sensor-based and traditional clinical metrics to assess upper-extremity impairment and function. The five common metrics included in the reviewed studies measured the number of velocity peaks (~ 9 studies), path-length ratio (~ 8 studies), the max speed of the arm (~ 7 studies), active range of motion (~ 7 studies), and movement time (~ 7 studies). The metrics are often compared to an established clinical assessment to determine validity of the metric. The sensor-based metrics can be categorized by the aspect in which they evaluate movement quality similar to De Los Reyes-Guzmán et al.: smoothness, efficiency, efficacy, accuracy, coordination, or range of motion [14]. Resistance, Movement Planning, Coordination, and Strength are included as additional categories since some of the reviewed sensor-based metrics best evaluate those movement aspects. Examples of common evaluation activities and specific metrics that have been computed to quantify movement quality are outlined in Table 2.

**Table 2:** Evaluation activities, measures, and metrics for both uni- and bi-manual assessment via upper-extremity robotic devices

Evaluation activity	Metrics	Measured aspect of movement quality
Point-to-point reaching or path following	Spectral [25], jerk [75], peaks [75, 76], minima speed count [10], amount of assistance [25], movement synergy [25], task time [25, 77, 78], accuracy [25], mean & peak speed [78], mean absolute value of the distance [75], path length ratio [75], active movement index [75], distance to path ratio [77], standard deviation on the target [77], reaction time [75]	Smoothness, efficacy, intra-limb coordination, efficiency, accuracy, movement planning
ADL: circle drawing and games	The axes ratio, joint angle correlation [78]	Intra- and inter-limb coordination, efficacy, efficiency
Arm position matching task	Standard deviation of the active hand's position, range of workspace matched by the active hand relative to the passive, mean of the mean error between the active and passive hands [77]	Inter-limb coordination
Joint and directional strength	Mean shoulder strength [78]	Strength
Resistance to passive movement assessment	Joint torque resistance [76]	Resistance to single joint to passive movement

### Smoothness

Lack of arm movement smoothness is a key indicator of underlying impairment [79]. Traditional therapist-administered assessments do not computationally measure smoothness leaving therapists unable to determine the degree to which disruption to movement smoothness is compromising motor function and, therefore, ADL. Most metrics that have been developed to quantify smoothness are based on features of the velocity profile of an arm movement, such as speed [80, 81], speed arc length [79], local minima of velocity [10], velocity peaks [75, 76, 81], tent [80], spectral [25], spectral arc length [25, 81], modified spectral arc length [79], and mean arrest period ratio [76]. Table 3 summarizes the smoothness metrics and their corresponding equations with equation numbers for reference. The speed metric is expressed as a ratio between the mean speed and the peak speed (Eq. 1). The speed arc length is the temporal length of the velocity profile (Eq. 2). Local minima of velocity and the velocity peaks metrics are measured by counting the number of minimum (Eq. 3) or maximum (Eq. 4) peaks in the velocity profile, respectively. The tent metric is a graphical approach that divides the area under the velocity curve by the area of a single peak velocity curve (Eq. 5). The spectral metric is the summation of the maximal Fourier transformed velocity vector (Eq. 6). The spectral arc-length metric is calculated from the frequency spectrum of the velocity profile by performing a fast Fourier transform operation and then computing the length (Eq. 7). The modified spectral arc length adapts the cutoff frequency according to a given threshold velocity and an upper-bound cutoff frequency (Eq. 8). The modified spectral arc length is then independent of temporal movement scaling. The mean arrest period ratio is the time portion that movement speed exceeds a given percentage of peak speed (Eq. 9).

**Table 3:** Velocity profile and jerk profile based smoothness metrics found in reviewed papers

		Metric	Description	Equation	Eqn
Velocity profile based metrics		Speed [80, 81]	Ratio of mean speed to peak speed	$\eta_{speed} = v_{mean} /v_{peak}$	(1)
		Speed arc length [79]	Temporal length of the velocity profile	$\eta_{spal} = - \ln \left( {\mathop \smallint \nolimits_{{t_{1} }}^{{t_{2} }} \sqrt {\left( {\frac{1}{{t_{2} - t_{1} }}} \right)^{2} + \left( {\frac{{d\hat{v}}}{dt}} \right)^{2} } dt} \right)$	(2)
		Local minima of velocity [10]	Number of minimums in the velocity profile	$\eta_{minima} = \sum Min\left( {v\left( t \right)} \right)$	(3)
		Velocity peaks [75, 76, 81]	Number of maximums in the velocity profile	$\eta_{peaks} = \sum Max\left( {v\left( t \right)} \right)$	(4)
		Tent [80]	Ratio of area under the entire velocity profile to area under a single-peak velocity profile	$\eta_{tent} = {{\mathop \smallint \nolimits_{{t_{1} }}^{{t_{2} }} v\left( t \right)dt} \mathord{\left/ {\vphantom {{\mathop \smallint \nolimits_{{t_{1} }}^{{t_{2} }} v\left( t \right)dt} {\mathop \smallint \nolimits_{{t_{1} }}^{{t_{2} }} v_{speak} \left( t \right)dt}}} \right. \kern-0pt} {\mathop \smallint \nolimits_{{t_{1} }}^{{t_{2} }} v_{speak} \left( t \right)dt}}$	(5)
		Spectral [25]	Summation of maxima Fourier transformed velocity vector	$Smoothness = - \sum Maxima_{{\overline{v}\left( \omega \right)}}$	(6)
		Spectral arc length [25, 81]	Vector norm of the frequency spectrum of the fast Fourier transformed velocity profile	$SAL = - \mathop \smallint \limits_{0}^{{\omega_{c} }} \sqrt {\left( {\frac{1}{{\omega_{c} }}} \right)^{2} + \left( {\frac{{d\hat{V}\left( \omega \right)}}{d\omega }} \right)^{2} } d\omega$	(7)
		Modified spectral arc length [79]	Spectral Arc Length with the cutoff frequency modified to a given threshold velocity and an upper-bound cutoff frequency	${\text{Eqn}}{\text{. 7 w/ }}\omega_{c} = \min \{ \omega_{c}^{\max } ,\min \left\{ {\omega ,\hat{V}\left( r \right)\left\langle {\overline{V} \forall r } \right\rangle \omega } \right\}\}$	(8)
		Mean arrest period ratio [76]	Time portion that movement speed exceeds a given percentage of peak speed	$\eta_{MAPR} = \frac{{t_{vc} }}{{t_{total} }}, vc \ge .1v_{peak}$	(9)
Jerk profile based metrics		Root mean square jerk [82]	Root-mean-square of the jerk normalized by the movement duration	$\eta_{rmsj} = - \sqrt {\frac{1}{{t_{2} - t_{1} }}\mathop \smallint \limits_{{t_{1} }}^{{t_{2} }} \left| {\frac{{d^{2} v}}{{dt^{2} }}} \right|^{2} dt}$	(10)
		Normalized mean absolute jerk [80, 82]	Mean of the magnitude jerk normalized or divided by the peak velocity	$\eta_{nmaJ} = - \frac{1}{{v_{peak} \left( {t_{2} - t_{1} } \right)}}\mathop \smallint \nolimits_{{t_{1} }}^{{t_{2} }} \left| {\frac{{d^{2} v}}{{dt^{2} }}} \right|dt$	(11)
		Dimensionless-squared jerk [80]	Square root of the integral of the square of the jerk times the duration of the movement to the fifth power over the length squared	$\eta_{dj} = - \frac{{\left( {t_{2} - t_{1} } \right)^{3} }}{{v_{peak}^{2} }}\mathop \smallint \limits_{{t_{1} }}^{{t_{2} }} \left| {\frac{{d^{2} v}}{dt}} \right|^{2} dt$	(12)
		Log dimensionless jerk [81]	Logarithm of normalized jerk defined in equation	$\eta_{ldj} = - \ln \left( {\frac{{\left( {t_{2} - t_{1} } \right)^{3} }}{{v_{peak}^{2} }}\mathop \smallint \limits_{{t_{1} }}^{{t_{2} }} \left| {\frac{{d^{2} v}}{dt}} \right|^{2} dt} \right)$	(13)

Equations have been rewritten using consistent variables where: $v\left( \omega \right)$ is the frequency domain of the limb’s velocity; $t_{i}$ is time at instant i; $v_{peak}$ is the peak velocity of the end-effector; $\omega_{c}$ is the cutoff frequency; $v_{speak}$ is the single peak velocity profile; $\hat{v}$ is the normalized velocity vector; $\overline{v}$ is the normalized zero-padded velocity vector; and $\overline{V}$ is a given threshold of the velocity in the frequency domain

Another commonly used approach is to analyze the jerk (i.e., the derivative of acceleration) profile. The common ways to assess smoothness using the jerk profile are root mean square jerk, mean rectified jerk, normalized jerk, and the logarithm of dimensionless jerk. The root mean square jerk takes the root-mean-square of the jerk that is then normalized by the movement duration [82] (Eq. 10). The mean rectified jerk (normalized mean absolute jerk) is the mean of the magnitude jerk normalized or divided by the peak velocity [80, 82] (Eq. 11). The normalized jerk (dimensionless-squared jerk) is the square of the jerk times the duration of the movement to the fifth power over the length squared (Eq. 12). It is then integrated over the duration and square rooted. The normalized jerk can be normalized by mean speed, max speed, or mean jerk [80]. The logarithm of dimensionless jerk (Eq. 13) is the logarithm of normalized jerk defined in Eq. 12 [81].

It has yet to be determined which smoothness metric is more effective for characterizing recovery of smooth movement. According to Rohrer et al. [80], the metrics of speed, local minima of velocity, peaks, tent, and mean arrest period ratio showed increases in smoothness for inpatient recovery from stroke, but the mean rectified jerk metric seemed to show a decrease in smoothness as survivors of stroke recovered. Rohrer et al. warned that a low smoothness factor in jerk does not always mean the person is highly impaired. The spectral arc-length metric showed a consistent increase in smoothness as the number of sub-movements decreased [25], whereas the other metrics showed sudden changes in smoothness. For example, the mean arrest period ratio and the speed metric showed an increase in smoothness with two or more sub-movements, but when two sub-movements started to merge, the smoothness decreased. As a result, the spectral arc-length metric appears to capture change over a wider range of movement conditions in recovery in comparison to other metrics.

### Resistance

The presence of a velocity-dependent hyperactive stretch reflex is referred to as spasticity [83]. Spasticity results in a lack of smoothness during both passive and active movements and is more pronounced with activities that involve simultaneous shoulder abduction loading and extension of the elbow, wrist, or fingers [83], which are unfortunately quite common in ADL. A standard approach to assessing spasticity by a therapist involves moving a subject’s passive arm at different velocities and checking for the level of resistance. While this manual approach is subjective, electronic sensors have the potential to assess severity of spasticity in much more objective ways. Centen et al. report a method to assess the spasticity of the elbow using an upper-limb exoskeleton [84] involving the measurement of peak velocity, final angle, and creep. Sin et al., similarly performed a comparison study between a therapist moving the arm versus a robot moving the arm. An EMG sensor was used to detect the catch and compared with a torque sensor to detect catch angle for the robotic motion [85]. The robot moving the arm seemed to perform better with the inclusion of either an EMG or a torque sensor than with the therapist moving the arm and the robot simply recording the movement. A related measure that may be correlated with spasticity is the assessment of joint resistance torques during passive movement [76]. This can provide an assessment of the velocity-dependent resistance to movement that arises following stroke.

### Efficiency

Efficiency measures movement fluency in terms of both task completion times and spatial trajectories. In point-to-point reaching, people who have suffered a stroke commonly display inefficient paths in comparison to their healthy side or compared to subjects who are unimpaired [10]. During the early phases of recovery after stroke, subjects may show slow overall movement speed resulting in longer task times. As recovery progresses, overall speed tends to increase and task times decrease, indicating more effective and efficient motor planning and path execution. Therapists usually observe the person’s efficiency in completing a task and then rate the person’s ability in completing a task in a timely manner. Therefore, both task time (or movement time) [10, 76, 77, 86, 87] and mean speed [25, 75, 77, 81, 86] are effective ways to assess temporal efficiency. Similar measures used by Wagner et al. include peak-hand velocity and time to peak-hand velocity [87]. To measure spatial efficiency of movement, both Colombo et al. [75], Mostafavi [77], and Germanotta [86] calculated the movement path length and divided it by the straight-line distance between the start and end points. This is known as the path-length ratio.

### Movement planning

Movement planning is associated with feedforward sensorimotor control, elements that occur before the initial phase of movement. A common approach is to use reaction time to assess the duration of the planning phase. In a typical clinical assessment, a therapist can only observe/quantify whether movement can be initiated or not, but has no way to quantify the lag between the signal to initiate movement and initiation of movement. Keller et al., Frisoli et al., and Mostafavi et al. quantified the reaction time to assess movement planning [10, 76, 77] in subjects who have suffered a stroke. Mostafavi assessed movement planning in three additional ways by assessing characteristics of the actual movement: change in direction, movement distance ratio, and maximum speed ratio [77]. The change in direction is the angular deviation between the initial movement vector and the straight line between the start and end points. The first-movement-distance ratio is the ratio between the distance the hand traveled during the initial movement and the total distance between start and end points. The first-movement-maximum speed ratio is the ratio of the maximum hand speed during the initial phase of the movement divided by the global hand speed for the entire movement task.

### Movement efficacy 

Movement efficacy measures the person’s ability to achieve the desired task without assistance. While therapists can assess the number of completed repetitions, they have no means to kinetically quantify amount of assistance required to perform a given task. Movement efficacy is quantified by robot sensor systems that can measure: (a) person-generated movement, and/or (b) the amount of work performed by the robot to complete the movement (e.g., when voluntary person-generated movement fails to achieve a target). Hence, movement efficacy can involve both kinematic and kinetic measures. A kinematic metric that can be used to represent movement efficacy is the active movement index, which is calculated by dividing the portion of the distance the person is to complete by the total target distance for the task [75]. An example metric based on kinetic data is the amount of assistance metric, proposed by Balasubramanian et al. [25]. It is calculated by estimating the work performed by the robot to assist voluntary movement, and then dividing it by the work performed by the robot as if the person performs the task without assistance from the robot. A similar metric obtained by Germanotta et al. calculates the total work by using the movement’s path length, but Germanotta et al. also calculate the work generated towards the target [86].

### Movement accuracy

**Fig. 4:**
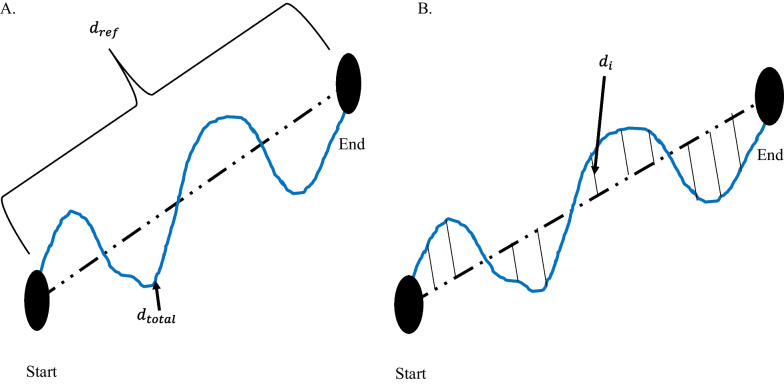
Difference between path-length ratio and mean absolute value of the distance. **A** Path-length ratio. $d_{ref}$ is the theoretical distance the hand should travel between the start and end point. $d_{total}$ is the total distance the hand travelled from Start to End. **B** Mean absolute value of the distance. $d_{i}$ is the distance between the theoretical path and the actual hand path

Movement accuracy has been characterized by the error in the end-effector trajectory compared to a theoretical trajectory. It measures the person’s ability to follow a prescribed path, whereas movement efficiency assesses the person’s ability to find the most ideal path to reach a target. Colombo et al. measured movement accuracy in people after stroke by calculating the mean-absolute value of the distance, which is the mean absolute value of the distance between each point on the person’s path and the theoretical path [75]. Figure 4 demonstrates the difference between path-length ratio and mean-absolute value of the distance. The mean-absolute value of the distance computes the error between a desired trajectory and the actual, and the path-length ratio computes the total path length the person’s limb has traveled. Another similar metric is the average inter-quartile range, which quantifies the average “spread” among several trajectories [15]. Balasubramanian et al. characterized movement accuracy as a measure of the subject’s ability to achieve a target during active reaching. They refer to the metric as movement synergy [25], and calculate it by finding the distance between the end-effector’s final location and the target location.

### Intra-limb coordination

Intra-limb (inter-joint) coordination is a measure of the level of coordination achieved by individual joints of a limb or between multiple joints of the same limb (i.e., joint synergy) when performing a task. Since the upper limb consists of kinematic redundancies, the human arm can achieve a desired outcome in multiple ways. For example, a person might choose to move an atypical joint in order to compensate for a loss of mobility in another joint. Frisoli et al. and Bosecker et al. used the shoulder and elbow angle to find a linear correlation between the two angles in a movement task that required multi-joint movement [10, 78]. In terms of clinical assessment, joint angle correlations can illustrate typical or atypical contribution of a joint while performing a multi-joint task.

### Inter-limb coordination

Inter-limb coordination refers to a person’s ability to appropriately perform bilateral movements with affected and unaffected arms. Therapists observe the affected limb by often comparing to the unaffected limb during a matching task, such as position matching. Matching can either be accomplished with both limbs moving simultaneously or sequentially, and typically without the use of vision. Dukelow et al. used position matching to obtain measures of inter-limb coordination [24], including trial-to-trial variability, spatial contraction/expansion, and systematic shifts. Trial-to-trial variability is the standard deviation of the matching hand’s position for each location in the x (distal/proximal), y (anterior/posterior), and both in x and y in the transverse plane. Spatial contraction/expansion is the ratio of the 2D work area of the target hand to the 2D work area of the matching hand during a matching task. Systematic shifts were found by calculating the mean absolute position error between the target and matching hand for each target location.

Semrau et al. analyzed the performance of subjects in their ability to match their unaffected arm with the location of their affected arm [88]. In the experiment, a robot moved the affected arm to a position and the person then mirrored the position with the unaffected side. The researchers compared the data when the person was able to see the driven limb versus when they were unable to see the driven limb. The initial direction error, path length ratio, response latency, peak speed ratio, and their variabilities were calculated to assess the performance of the person’s ability to perform the task.

### Range of motion

Range of motion is a measure of the extent of mobility in one or multiple joints. Traditionally, range of motion can be measured with the use of a goniometer [89]. The goniometer measures the individual joint range of motion, which takes considerable time. Range of motion can be expressed as a 1-DOF angular measure [76, 89], a 2-DOF planar measure (i.e., work area) [82], or a 3-DOF spatial measure (i.e., workspace) [77]. Individual joints are commonly measured in joint space, whereas measures of area or volume are typically given in Cartesian space. In performing an assessment of work area or workspace with a robotic device, the measure can be estimated either by: (a) measuring individual joint angles with an exoskeleton device and then using these angles to compute the region swept out by the hand, or (b) directly measuring the hand or fingertips with a Cartesian (end-effector) device. The measurement of individual joint range of motion (ROM) as well as overall workspace have significant clinical importance in assessing both passive (pROM) and active (aROM) range of motion. To measure pROM, the robot drives arm movement while the person remains passive. The pROM is the maximum range of motion the person has with minimal or no pain. For aROM, a robot may place the arm in an initial position/orientation from which the person performs unassisted joint movements to determine the ROM of particular joints [76], or the area or volume swept by multiple joints. Lin et al. quantified the work area of the elbow and shoulder using potentiometers and derived test–retest reliability [89]. The potentiometer measurements were then compared to therapist measurements to determine validity.

### Strength

Measures of strength evaluate a person’s ability to generate a force in a direction or a torque about a joint. Strength measurements may involve single or multiple joints. At the individual joint level, strength is typically measured from a predefined position of a person’s arm and/or hand. The person then applies a contraction to produce a torque at the assessed joint [76, 78]. Multi-joint strength may also be measured by assessing strength and/or torque in various directions at distal locations along the arm, such as the hand. Lin et al. compared the grip strength obtained from load cells to a clinical method using precise weights, which showed excellent concurrent validity [89].

## Measures and methods based on neural activity using EEG/EMG

Although much information can be captured and analyzed using the kinematic and kinetic measures listed above, their purview is limited. These measures provide insight into the functional outcomes of neurological system performance but provide limited perspective on potential contributing sources of measured impairment [90]. For a deeper look into the neuromuscular system, measures based on neurological activation are often pursued. As a complement to biomechanical measures, methods based on quantization of neural activity like EEG and EMG have been used to characterize the impact of stroke and its underlying mechanisms of impairments [91, 92]. Over the past 20 years, numerous academic research studies have used these measures to explore the effects of stroke, therapeutic interventions, or time on the evolution of abnormal neural activity [91]. Groups with different levels of neurological health are commonly compared (e.g., chronic/acute/subacute stroke vs. non-impaired, or impairment level) or other specific experimental characteristics (e.g., different rehabilitation paradigms [93, 94]). With this evidence, the validity of these metrics has been tested; however, the study of reliability of these metrics is needed to complete the jump from academic to clinical settings.

Extracting biomarkers from non-invasive neural activity requires careful decomposition and processing of raw EEG and EMG recordings [32]. Various methods have been used, and the results have produced a growing body of evidence for the validity of these biomarkers in providing insight on the current and future state of motor, cognitive, and language skills in people after stroke [38, 95]. Some of the biomarkers derived from EEG signals include: power-related band-specific information [34, 35, 43, 47, 53, 54, 96–101], band frequency event-related synchronization and desynchronization (ERS/ERD) [22, 51, 102, 103], intra-cortical coherence or functional connectivity [39, 59, 73, 94, 104–109], corticomuscular coherence (CMC) [37, 110–113], among others [114, 115]. Biomarkers extracted from EEG can be used to assess residual functional ability [38, 54, 73, 97–99], derive prognostic indicators [34, 43, 104], or categorize people into groups (e.g., to better match impairments with therapeutic strategies) [39, 47, 58, 116].

In the following subsections, valid biomarkers derived mostly from EEG signal features (relationship with motor outcome for a person after stroke) will be discussed and introduced theoretically. Distinctions will be made about the stage after stroke when signals were taken. Findings are reported from 33 studies that have examined the relationship between extracted neural features and motor function for different groups of people after stroke. These records are grouped by quantization methods used including approaches based on measures of frequency spectrum power (n = 9), inter-regional coherence (n = 10 for cortical coherence and n = 9 for CMC), and reliability (n = 5).

### Frequency spectrum power

Power measures the amount of activity within a signal that occurs at a specific frequency or range of frequencies. Power can be computed in absolute or relative terms (i.e., with respect to other signals). It is often displayed as a power density spectrum where the magnitudes of signal power can be seen across a range of frequencies. In electro-cognitive research, the representation of power within specific frequency bands has been useful to explain brain activity and to characterize abnormal oscillatory activity due to regional neurological damage [32, 117].

#### Frequency bands in EEG content

Electrical activity in the brain is dominated primarily by frequencies from 0–100 Hz where different frequency bands correspond with different states of activity: Delta (0–4 Hz) is associated with deep sleep, Theta (4–8 Hz) with drowsiness, Alpha (8–13 Hz) with relaxed alertness and important motor activity [117], and Beta (13–31 Hz) with focused alertness. Gamma waves (> 32 Hz) are also seen in EEG activity; however, their specific relationship to level of alertness or consciousness is still debated [32, 117]. Important cognitive tasks have been found to trigger activity in these bands in different ways. Levels of both Alpha and Delta activity have also been shown to be affected by stroke and can therefore be examined as indicators of prognosis or impairment in sub-acute and chronic stroke [52, 100, 118].

#### Power in acute and sub-acute stroke

For individuals in the early post-stroke (i.e., sub-acute) phase, abnormal power levels can be an indicator of neurological damage [98]. Attenuation of activity in Alpha and Beta bands have been observed in the first hours after stroke [100] preceding the appearance of abnormally high Delta activity. Tolonen et al. reported a high correlation between Delta power and regional Cerebral Blood Flow (rCBF). This relationship appears during the sub-acute stroke phase and has been used to predict clinical, cognitive, and functional outcomes [119]. Delta activity has also been shown to positively correlate with 1-month National Institutes of Health Stroke Scale (NIHSS) [52] and 3-month Rankin scale [36] assessments.

Based on these findings, several QEEG (Quantitative Electroencephalography) metrics involving ratios of abnormal slow (Delta) and abnormal fast (Alpha and Beta) activity have been developed. The Delta-Alpha Ratio (DAR), Delta-Theta Ratio (DTR), and (Delta + Theta)/(Alpha + Beta) Ratio (DTABR also known as PRI for Power Ratio Index) relate amount of abnormal slow activity with the activity from faster bands and have been shown to provide valuable insight into prognosis of stroke outcome and thrombolytic therapy monitoring [98]. Increased DAR and DTABR have been repeatedly found to be the QEEG indices that best predict worse outcome for the following: comparing with the Functional Independence Measure and Functional Assessment Measure (FIM-FAM) at 105 days [53], Montreal Cognitive Assessment (MoCa) at 90 days [54], NIHSS at 1 month [35], modified ranking scale (mRS) at 6 months [105], NIHSS evolution at multiple times [120], and NIHSS at 12 months [96]. DAR was also used to classify people in the acute phase and healthy subjects with an accuracy of 100% [58].

The ability of basic EEG monitoring to derive useful metrics during the early stage of stroke has made EEG collection desirable for people who have suffered a stroke in intensive care settings. The derived QEEG indices have proven to be helpful to determine Delayed Cerebral Ischemia (DCI), increased DAR [43], and increased Delta power [34, 118]. However, finding the electrode montage with the least number of electrodes that still reveals the necessary information for prognoses is one of the biggest challenges for this particular use of EEG. Comparing DAR from 19 electrodes on the scalp with 4 electrodes on the frontal cortex suggests that DAR from 4 frontal electrodes may be enough to detect early cognitive and functional deficits [53]. Studies explored the possibility of a single-electrode montage over the Fronto-Parietal area (FP1); the DAR and DTR from this electrode might be a valid predictor of cognitive function after stroke when correlated with the MoCA [54], relative power in Theta band correlated with mRS and modified Barthel Index (mBI) 30 and 90 days after stroke [121].

#### Power in chronic stroke

The role of power-related QEEG indices during chronic stroke and progression of motor functional performance have been examined with respect to rehabilitation therapies, since participants have recovered their motion to a certain degree [4]. Studies have shown that therapy and functional activity improvements correlate with changes of the shape and delay of event-related desynchronization and synchronization (ERD-ERS) for time–frequency power features when analyzing Alpha and Beta bands on the primary motor cortex for ipsilesional and contralesional hemispheres [21, 22, 122]. Therapies with better outcome tend to have reduced Delta rhythms and increased Alpha rhythms [122].

Bertolucci [47] compared starting power spectrum density in different bands for both hemispheres with changes in WMFT and FMA over time. Increased global Alpha and Beta activity was shown to correlate with better WMFT evolution while, increase in contralesional Beta activity was shown to be correlated with FMA evolution. Metrics combining slow and fast activity have also been tested in the chronic stage of stroke, significant negative correlation between DTABR (PRI) at the start of therapy was related to FMA change during robotic therapy [99]. This finding suggests that DTABR may have promise as prognostic indicators for all stages of stroke.

Brain Symmetry Index (BSI) is a generalized measure of “left to right” (affected to non-affected) power symmetry of mean spectral power per hemisphere. These inter-hemispheric relationships of power have been used as prognostic measures during all stages of stroke. Baseline BSI (during the sub-acute stage) was found to correlate with the FMA at 2 months [73], mRS at 6 months [123], and FM-UE predictor when using only theta band BSI for patients in the chronic stage [124]. BSI can be modified to account for the direction of asymmetry, the directed BSI at Delta and Theta bands proved meaningful to describe evolution from acute to chronic stages of upper limb impairment as measured by FM-UE [120, 125]. Table 4 and Table 11 in Appendix 1 communicate power-derived metrics across different stages of stroke documented in this section and their main reported relationships with motor function. Findings are often reported in terms of correlation with clinical tests of motor function.

**Table 4:** Summary of EEG signal power metrics and relationship to motor function or outcome in stroke

Metric	Stage	Correlation with clinical measures/findings [references]
Global Beta power Global Alpha power	Acute	First noticeable electrical changes during stroke [58], 105-day FIM FAM [53], 12-months NIHSS [96]
Delta power, Theta power	Sub-Acute	1-month NIHSS [52], 30 and 90 days mBI and mRS [121]
Delta-Alpha Ratio (DAR) Delta-Theta Ratio (DTR) (Delta + Theta)/(Alpha + Beta) Ratio (DTABR)	Sub-Acute	cognitive deficit [53], 90-days MoCA [54], 6-months mRS [105], 12 months NIHSS[96], 6-months FIM FAM [97], NIHSS evolution 3 weeks to 6-months [120]
Brain Symmetry Index (BSI) Pairwise derived brain symmetry index (pdBSI)	Sub-Acute	2-months FMA [104]*, 6-months mRS [125]
Global Delta	Chronic	motor therapy gains [122]
Delta-Alpha Ratio (DAR) Power Ratio Index (PRI)	Chronic	FMA evolution [99] No relation with FM-UE [125]
Brain Symmetry Index (BSI)	Chronic	FM-UE [125], directional BSI delta band correlated with FM-UE improvement and NIHSS evolution [120]

An extended version of this table that includes demographic information can be found in Table 11 in Appendix 1

*Records added manually

### Brain connectivity (cortical coherence)

Brain connectivity is a measure of interaction and synchronization between distributed networks of the brain and allows for a clearer understanding of brain function. Although cortical damage from ischemic stroke is focal, cortical coherence can explain abnormalities in functionality of remote zones that share functional connections to the stroke-affected zone [59].

Several estimators of connectivity have been proposed in the literature. Coherency, partial coherence (pCoh) [125], multiple coherence (mCoh), imaginary part of coherence (iCoh) [126], Phase Lagged Index (PLI), weighted Phase Lagged Index (wPLI) [127], and simple ratios of power at certain frequency bands [73] describe synchronic symmetric activity between ROIs and are referred to as non-directed or functional connectivity [128]. Estimators based on Granger’s prediction such as partial directed coherence (PDC) [129–131], or directed transfer Function (DTF) [132, 133] and any of their normalizations describe causal relationships between variables and are referred to as directed or effective connectivity [134]. Connectivity also allows the analysis of brain activity as network topologies, borrowing methods from graph theory [32, 134]. Network features such as complexity, linearity, efficiency, clustering, path length, node hubs, and more can be derived from graphs [128]. Comparisons of these network features among groups with impairment and healthy controls have proven to be interesting tools to understand and characterize motor and functional deficits after stroke [108].

Studies have used intra- and inter-cortical coherence to expand the clinical understanding of the neural reorganization process [59, 106–109], as a clinical motor and cognitive predictor [38, 94, 104, 135, 136], and as a tool to predict the efficacy of rehabilitation therapy [94]. Table 5 and Table 12 in Appendix 2 briefly summarize the main metrics discussed in this section and their results that are related with motor function assessment. In general, studies have shown that motor deficits in stroke survivors are related to less connectivity to main sensory motor areas [38, 94, 104, 137], weak interhemispheric sensorimotor connectivity [109, 138], less efficient networks [106, 135], with less “small world” network patterns [108, 134] (small-world networks are optimized to integrate specialized processes in the whole network and are known as an important feature of healthy brain networks).

**Table 5:** Summary of metrics from EEG brain connectivity and main findings related to motor function

Metric	Stage	Correlation with clinical measures/findings [reference]
Delta and Alpha2 small-worldness	Acute	Significant differences between people with stroke and controls [108]*
Maximum coherence PLI alpha band	Sub-Acute	Predictor of FM-UE score [139]
Ipsilesional High beta M1-PM and network to M1 coherence	Chronic	Motor therapy gains, FMA predictor [94], negative correlation with FMA evolution over a month
Interhemispheric M1 Beta	Chronic	PDC with FMA-UE and hand recovery [109], dwPLI with ARAT and FM-UE [138]
Motor cortex Weighted node Degree (WND) from iCoh beta band	Chronic	FMA-UE, Nine Hole Peg Test [38]
Alpha connectivity with motor cortex	Chronic	3-months FMA [104]*
Beta, Gamma Normalized inter hemispheric strength (nIHS) from PDC	Chronic	Cortico Spinal Tract (CST) integrity, impairment [136]
Global and local Beta/Gamma bands network efficiency	Chronic	Survivors of stroke have less-efficient networks vs. unimpaired [106]*, FMA-UE [135]

An extended version of this table that includes demographic information can be found in Table 12 in Appendix 2

*Record added manually

Survivors of stroke tend to exhibit more modular (i.e., more clustered, less integrated) and less efficient networks than non-impaired controls with the biggest difference occurring in the Beta and Gamma bands [106]. Modular networks are less “small-world” [134]; small-world networks are optimized to integrate specialized processes in the whole network and are known as an important feature of healthy brain networks. Such a transition to a less small-world network was observed during the acute stage of stroke (first hours after stroke) and documented to be bilaterally decreased in the Delta band and bilaterally increased in the high Alpha band (also known as Alpha2: 10.5–13 Hz) [108].

Global connectivity with the ipsilesional primary motor cortex (M1) is the most researched biomarker derived from connectivity and has been studied in longitudinal experiments as a plasticity indicator leading to future outcome improvement [38], motor and therapy gains [94], upper limb gains during the sub-acute stage [137], and as a feature that characterizes stroke survivors’ cognitive deficits [104]. Pietro [38] used iCoh to test the weighted node degree (WND), a measure that quantifies the importance of a ROI in the brain, for M1 and reported that Beta-band features are linearly related with motor improvement as measured by FM-UE and Nine-Hole-Peg Test. Beta-band connectivity to ipsilesional M1, as measured by spectral coherence, can be used as a therapy outcome predictor, and more than that, results point heavily toward connectivity between M1 and ipsilesional frontal premotor area (PM) to be the most important variable as a therapy gain predictor; predictions can be further improved by using lesion-related information such as CST or MRI to yield more accurate results [94]. Comparisons between groups of people with impairment and controls showed significant differences on Alpha connectivity involving ipsilesional M1, this value showed a relation with FMA 3 months for the group with impairment due to stroke [104].

The relationship between interhemispheric ROI connectivity and motor impairment has been studied. The normalized interhemispheric strength (nIHS) from PDC was used to quantify the coupling between structures in the brain, Beta- and lower Gamma-band features of this quantity in sensorimotor areas exhibited linear relationships with the degree of motor impairment measured by CST [136]. A similar measure, also derived from PDC used to measure ROI interhemispheric importance named EEG-PDC was used in [109]; here the results show that Mu-band (10–12 Hz) and Beta-band features could be used to explain results for hand motor function from FM-UE. In another study, Beta debiased weighted phase lag index (dwPLI), correlated with outcome measured by Action Research Arm Test (ARAT) and FM-UE [138].

Global and local network efficiency for Beta and Gamma bands seem to be significantly decreased in the population who suffered from a stroke compared to healthy controls as reported in [106]. Newer results, such as the ones pointed out by [135] found statistically significant relationships between Beta network efficiency, network intradensity derived using a non-parametric method (named Generalized Measure of Association), and functional recovery results given by FM-UE. Global maximal coherence features in the Alpha band have been recently recognized as FM-UE predictors, where coherence was computed using PLI and related to motor outcome by means of linear regression [139].

### *Corticomuscular coherence*

Corticomuscular coherence (CMC) is a measure of the amount of synchronous activity between signals in the brain (i.e., EEG or MEG) and associated musculature (i.e., EMG) of the body [92]. Typically measured during voluntary contractions [110], the presence of coherence demonstrates a direct relationship between cortical rhythms in the efferent motor commands and the discharge of neurons in the motor cortex [140]. CMC is computed as correlation between EEG and EMG signals at a given frequency. Early CMC research found synchronous (correlated) activity in Beta and low Gamma bands [40–42]. CMC is strongest in the contralateral motor cortex [141]. This metric seems to be affected by stroke-related lesions, and thus provides an interesting tool to assess motor recovery [111, 142–144]. The level of CMC is lower in the chronic stage of stroke than in healthy subjects [112, 145], with chronic stroke survivors showing lower peak CMC frequency [146], and topographical patterns that are more widespread than in healthy people; highlighting a connection to muscle synergies [142, 147, 148]. CMC has been shown to increase with training [37, 112, 144].

Corticomuscular coherence has been proposed as a tool to: (a) identify the functional contribution of reorganized cortical areas to motor recovery [37, 112, 141, 144, 146]; (b) understand functional remapping [93, 142, 145]; and (c) study the mechanisms underlying synergies [147, 148]. CMC has shown increased abnormal correlation with deltoid EMG during elbow flexion for people who have motor impairment [147], and the best muscles to target with rehabilitative interventions [148]. Changes in CMC have been shown to correlate with motor improvement for different stages of stroke, although follow-up scores based on CMC have not shown statistically significant correlations when compared to clinical metrics [37, 93]. Results summarizing CMC on stroke can be found in Table 6 and Table 13 in Appendix 3.

**Table 6:** Summary of metrics from EEG-EMG coherence and main findings related to motor function

Metric	Stage	Correlation with clinical measures/findings [reference]
Beta, Gamma CMC	Chronic	Flexion synergy [93], FMA [37]
Beta CMC	Sub-acute	Function recovery, FMA-UE [141]
Gamma interhemispheric disparity	Chronic	Compensation of healthy limb [145]
CMC frequency peak	Acute, chronic	Characterization of CMC from stage to stage [146], non-significant differences among stages [112], Beta peaks related to co-contraction [149]
CMC topographical patterns	Acute, sub-acute	FMA, FMA-UE [144], level of impairment [142]

An extended version of this table that includes demographic information can be found in Table 13 in Appendix 3

## Reliability of measures

Each of the aforementioned measures have the potential to be integrated into robotic devices for upper-limb assessment. However, to improve the clinical acceptability of robotic-assisted assessment, the measurements and derived metrics must meet reliability standards in a clinical setting [55]. Reliability can be defined as the degree of consistency between measurements or the degree to which a measurement is free of error. A common method to represent the relative reliability of a measurement process is the intraclass correlation coefficient (ICC) [150]. Koo and Li suggest a guideline on reporting ICC values for reliability that includes the ICC value, analysis model (one-way random effects, two-way random effects, two-way fixed effects, or two-way mixed effects), the model type per Shrout and Fleiss (individual trials or mean of k trials), model definition (absolute agreement or consistency), and confidence interval [68]. Koo and Li also provide a flowchart in selecting the appropriate ICC based on the type of reliability and rater information. An ICC value below 0.5 indicates poor reliability, 0.5 to 0.75 moderate reliability, 0.75 to 0.9 good reliability, and above 0.9 excellent reliability. The reviewed papers will be evaluated based on these guidelines. For reporting the ICC, the Shrout and Fleiss convention is used [68]. The chosen reliability studies are included in the tables if the chosen ICC model, type, definition, and confidence interval are identifiable, and the metrics have previously been used in electronic-based metrics. For studies that report multiple ICC scores due to assessment of test–retest reliability for multiple raters, the lowest ICC reported is included to avoid bias in the reported results.

**Fig. 5:**
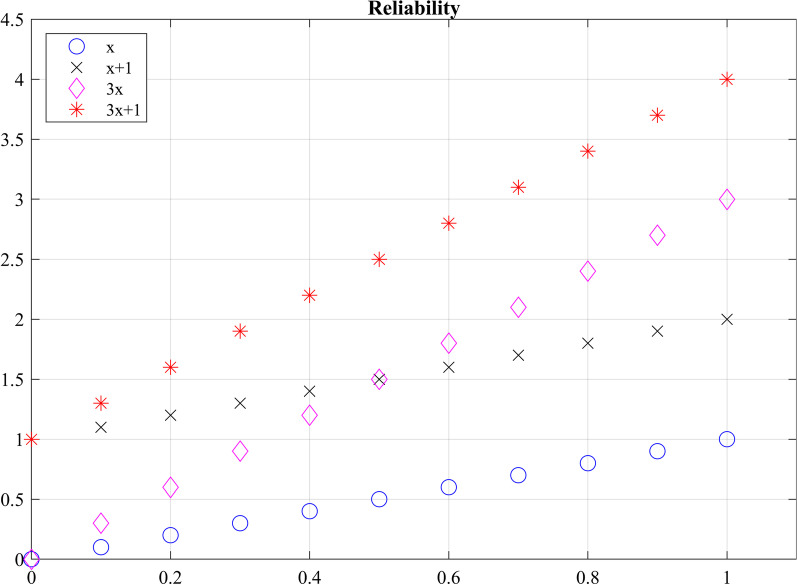
Checking agreement versus consistency among ratings. For y = x, the absolute ICC score is 1 and the consistency ICC score is 1.00. For y = x + 1, the agreement ICC score is 0.18 and the consistency ICC score is 1.00. For y = 3x, the absolute ICC score is 0.32 and the consistency ICC score is 0.60. For y = 3x + 1, the absolute ICC score is 0.13 and the consistency ICC score is 0.60

In the assessment of reliability of data from robotic sensors, common ways to assess reliability are to correlate multiple measurements in a single session (intra-session) and correlate multiple measurements between different sessions (inter-session) measurements (i.e., test–retest reliability) [151]. Checking for test–retest reliability determines the repeatability of the robotic metric. The repeatability is the ability to reproduce the same measurements under the same conditions. Table 7 shows the test–retest reliability of several robotic metrics. For metrics checking for test–retest reliability, a two-way mixed-effects model with either single or multiple measurements may be used [68]. Since the same set of sensors will be used to assess subjects, the two-way mixed model is used. The test–retest reliability should be checking for absolute agreement. Checking for absolute agreement (y = x) rather than consistency (y = x + b) determines the reliability without a bias or systematic error. For example, in Fig. 5, for a two-way random effect with a single measurement checking for agreement gives a score of 0.18. When checking for consistency, the ICC score reaches to 1.00. In other words, the bias has no effect on the ICC score when checking for consistency. Therefore, when performing test–retest reliability, it is important to check for absolute agreement to prevent bias in the test–retest result.

**Table 7:** Repeatability for sensor-based metrics

Metric	ICC(r,k)	Score (p < 0.05)	Rating	Measurement device	Session	Ref.
Isokinetic motion with EMG	ICC(2,1)	(0.857–0.981)	***-****	1-DOF	Intra-	[85]
Isokinetic motion with torque	ICC(2,1)	(0.873–0.987)	***-****			
Manual motion with EMG	ICC(2,1)	(0.538–0.924)	**-****			
Joint ROM right shoulder	ICC(3,1)	0.998	****	2-DOF (unactuated)	Intra-	[89]
Joint ROM right elbow	ICC(3,1)	0.994	****			
Joint ROM left shoulder	ICC(3,1)	0.996	****			
Joint ROM left elbow	ICC(3,1)	0.994	****			
Grip force right dynamometer	ICC(3,1)	0.998	****			
Grip force left dynamometer	ICC(3,1)	0.998	****			
Mean speed	ICC(3,1)	0.95, 0.93	****	MEMOS & Braccio di Ferro	Intra-, Inter-	[151]
Mean absolute value of the distance	ICC(3,1)	0.91, 0.97	****			
Normal path length	ICC(3,1)	0.9, 0.96	****			
Ratio between peak tangential speed and mean speed	ICC(3,1)	0.99, 0.91	****			
Spectral arc length	ICC(3,1)	0.92, 0.95	****			
Velocity peaks	ICC(3,1)	0.85, 0.95	***,****			
Mean speed	ICC(3,1)	0.93,0.94^a^	****	Kinect	Intra-, Inter-	[81]
Normalized mean speed	ICC(3,1)	0.81,0.6^a^	***,**			
Normalized speed peaks	ICC(3,1)	0.77,0.71^a^	***,**			
Logarithm of dimensionless jerk	ICC(3,1)	0.91,0.95^a^	****			
Curvature	ICC(3,1)	0.91,0.96^a^	****			
Spectral arc length	ICC(3,1)	0.52,0.12^a^	**,*			
Shoulder angle	ICC(3,1)	0.99,0.96^a^	****			
Elbow angle	ICC(3,1)	0.94,0.92^a^	****			
Duration	ICC(2,k)	0.962	****	MOTORE	Inter-	[86]
Mean speed	ICC(2,k)	0.914	****			
The total length of the path traveled	ICC(2,k)	0.951	****			
Mean of path length ratios	ICC(2,k)	0.972	****			
Line integral of force along patient path (Work_tot_)	ICC(2,k)	0.908	****			
Total work directed towards the target (Work_tan_)	ICC(2,k)	0.957	****			
Movement time	ICC(2,k)	0.11–0.82	*-***	7-camera Qualisys Motion	Inter-	[87]
Peak hand velocity	ICC(2,k)	0.74–0.95	**-****			
Time to peak hand velocity	ICC(2,k)	0.11–0.83	*-***			
Reach path ratio	ICC(2,k)	0.33–0.95	*-****			
Endpoint error	ICC(2,k)	0.68–0.85	**-***			
Reach extent	ICC(2,k)	0.93–0.99	****			
Maximum shoulder flexion range of motion	ICC(2,k)	0.93–0.95	****			
Maximum shoulder abduction ROM	ICC(2,k)	0.58–0.77	**-***			
Min elbow extension ROM	ICC(2,k)	0.86–0.91	***-****			
Interjoint coordination	ICC(2,k)	0.66–0.92	**-****			
Trajectory smoothness	ICC(2,k)	0.43–0.84	*-***			
aROM elbow flexion	ICC(3,k)	0.56–0.90	**-****	Inertial sensors	Rater A	[152]
aROM elbow pronation	ICC(3,k)	0.71–0.93	**-****			
aROM elbow supination	ICC(3,k)	0.84–0.97	***-****			
aROM wrist flexion	ICC(3,k)	0.86–0.97	***-****			
aROM wrist extension	ICC(3,k)	0.62–0.92	**-****			
aROM radial deviation	ICC(3,k)	0.63–0.92	**-****			
aROM ulnar deviation	ICC(3,k)	0.72–0.94	**-****			
Discrimination threshold	ICC(3,1)	0.52–0.90	**-****	KINARM		[157]
Displacement variability	ICC(3,1)	0.28–0.86	*-***			
Absolute error-XY	ICC(3,1)	0.74–0.95	**-****			
Variability-XY	ICC(3,1)	− 0.09–0.63	*-**			
Con/Exp ratio-XY	ICC(3,1)	0.68–0.94	**-****			
Spatial shift-XY	ICC(3,1)	0.72–0.95	**-****			
Visually guided reaching—dominate	ICC(3,1)	0.3	*			[158]
Visually guided reaching—non-dominate	ICC(3,1)	0.33	*			
Arm position matching—dominate	ICC(3,1)	0.29	*			
Arm position matching—non-dominate	ICC(3,1)	0.36	*			
Total movement time	ICC(2,k)	0.91–0.99	****	ArmeoSpring	Inter-	[62]
Movement time	ICC(2,k)	0.47–0.90	*-****			
Peak velocity	ICC(2,k)	0.83–0.97	***-****			
Hand path ratio	ICC(2,k)	0.47–0.90	*-****			
Velocity peaks	ICC(2,k)	0.45–0.91	*-****			
Score	ICC(2,k)	0.97–0.99	****			

*ICC(2,1)* two-way random effects with a single measurement, *ICC(2,k)* two-way random effects with a mean of k measurements, *ICC(3,1)* two-way mixed effects with a single measurement, *ICC(3,k)* two-way mixed effects with a mean of k measurements. *Poor reliability (< 0.5). **Moderate reliability (0.5 ≤ ICC < 0.75). ***Good reliability (0.75 ≤ ICC < 0.9). ****Excellent reliability (≥ 0.9)

^a^p value not reported

^***^,****Refers to intra- and inter-session reliability rating

^***^-**** is between good and excellent reliability based on the confidence interval

Not only should a robotic metric demonstrate repeatability, it should also be reproducible when different operators are using the same device. Reproducibility evaluates the change in measurements when conditions have changed. Inter-rater reliability tests have been performed to determine the effect raters have when collecting measurements when two or more raters perform the same experimental protocol [68]. To prevent a biased result, raters should have no knowledge of the evaluations given by other raters, ensuring that raters’ measurements are independent from one another. Table 8 shows the reproducibility of several robotic biomechanical metrics. All the included studies have used two raters to check for reproducibility. The researchers performed a two-way random effects analysis with either a single measurement or multiple measurements to check for agreement.

**Table 8:** Reproducibility table

Metric	ICC(r,k)	Score (p < 0.05)	Rating	Measurement device	Notes	Refs.
Isometric joint torque	ICC(3,1)	0.80–0.98 (p < 0.01)	***-****	ARMin		[76]
Isokinetic motion with EMG	ICC(2,1)	0.890 (0.685–0.961)	**-****	1-DOF		[85]
Isokinetic motion with torque	ICC(2,1)	0.931 (0.791–0.978)	***-****			
Manual motion with EMG	ICC(2,1)	0.788 (0.493–0.920)	*-****			
Initial direction error	ICC(2,k)	0.81, 0.95	***,****	KINARM	No vision, vision	[88]
Initial direction error variability	ICC(2,k)	0.84, 0.94	***,****			
Path length ratio	ICC(2,k)	0.68, 0.44	**,*			
Path length ratio variability	ICC(2,k)	0.95, 0.97	****			
Response latency	ICC(2,k)	0.92, 0.94	****			
Response latency variability	ICC(2,k)	0.91, 0.95	****			
Peak speed ratio	ICC(2,k)	0.71, 0.96	**,****			
Peak speed ratio variability	ICC(2,k)	0.86, 0.66	***,**			
Peak velocity	ICC^u^	0.9	****		Extension	[84]
Final angle	ICC^u^	0.86	***			
Creep	ICC^u^	0.66	**			
Peak velocity	ICC^u^	0.95	****		Extension difference	
Final angle	ICC^u^	0.91	****			
Creep	ICC^u^	0.74	**			
Peak velocity	ICC^u^	0.87	***		Flexion	
Final angle	ICC^u^	0.84	***			
Creep	ICC^u^	0.86	***			
Peak velocity	ICC^u^	0.88	***		Flexion difference	
Final angle	ICC^u^	0.88	***			
Creep	ICC^u^	0.79	***			
Var_xy	ICC^u^	0.81	***			[24]
Cont/exp_xy	ICC^u^	0.86	***			
Shift_xy	ICC^u^	0.7	**			
aROM elbow flexion	ICC(2,k)	0.89–0.97	***-****	Inertial sensors	First session	[152]
aROM elbow pronation	ICC(2,k)	0.89–0.94	***-****			
aROM elbow supination	ICC(2,k)	0.91–0.98	****			
aROM wrist flexion	ICC(2,k)	0.94–0.99	****			
aROM wrist extension	ICC(2,k)	0.86–0.97	***-****			
aROM radial deviation	ICC(2,k)	0.92–0.98	****			
aROM ulnar deviation	ICC(2,k)	0.90–0.98	****			

ICC^u^ – Unable to determine

ICC(2,1)—Two-way random effects with a single measurement. ICC(2,k)—Two-way random effects with a mean of k measurements. ICC(3,1)—Two-way mixed effects with a single measurement. ICC(3,k)—Two-way mixed effects with a mean of k measurements. *Poor reliability (< 0.5). **Moderate reliability (0.5 ≤ ICC < 0.75). ***Good reliability (0.75 ≤ ICC < 0.9). ****Excellent reliability (≥ 0.9)

### Measurement reliability of robotic biomechanical assessment

Of the 24 papers reviewed for biomechanical metrics, 13 papers reported on reliability. 6 papers reported reproducibility and 9 papers reported on repeatability. Overall, the metrics seem to demonstrate good to moderate reliability for both repeatability and reproducibility. However, caution should be exercised in determining which robotic metric is more effective in assessing movement quality based on reliability studies. The quality of measurements is highly dependent on the quality of the robotic device and sensors [85]. Having a completely transparent robot with a sensitive and accurate sensor will further improve assessment of reliability. Also, the researchers have used different versions of the ICC, as seen in Tables 7 and 8, which complicates direct comparisons of the metrics.

### Reliability of electrophysiological signal features

Of the 33 papers reviewed for electrophysiological metrics, 5 papers reported on reliability. 6 papers reported on repeatability. Convenience of acquiring electrophysiological signals non-invasively is relatively new. Metrics for assessment of upper limb motor impairment in stroke, derived from these signals have shown to be valid in academic settings, but most of these valid metrics have yet to be tested for intra- and inter-session reliability to be used in clinical and rehabilitation settings. Few studies found as a result of our systematic search have looked at test–retest reliability of these metrics. Therefore, we found and manually added records reporting on intra- and inter-session reliability on metrics based on electrophysiological features described in section “Measures and methods based on neural activity using EEG/EMG”, even if reliability was not assessed on people with stroke. Relevant results are illustrated in Table 9.

**Table 9:** Test–retest reliability of electrophysiological signal features for frequency bands of interest

Metric	ICC(r,k)	Band Score (p < 0.05)						#Ch	Method	Notes	Refs.
		Delta	Theta	Alpha	Beta	Gamma	Global				
Average clustering coefficient	(3,1)	0.59	**0.91**	**0.84, 0.87**	**0.73**	0.62	–	32	PLI	Infant subjects Alpha = Alpha1, Alpha2 Alpha1: (6–9) Hz, Alpha2: (9–12) Hz	[60]^
Path length		0.53	**0.89**	**0.84, 0.84**	**0.72**	0.59	–	32			
Small‐worldness index		0.25	0.56	0.67, 0.21	0.14	0.13	–	32			
Global connectivity		**0.60** − 0.29	**0.91** **0.82**	**0.84, 0.86** **0.75, 0.91**	**0.72** **0.74**	0.61 0.49	– –	32	PLI dwPLI		
Global connectivity	(1,1)	0.09, -0.07	0.06, 0.29	**0.64, 0.95**	0.32, 0.52	0.32 0.5	–	256	iCoh	5, 30 trials	[156] ^
		− 0.04, − 0.1	0.11 0.24	0.48, **0.91**	0.32, **0.65**	0.4, 0.62	–	256	wPLI	5, 30 trials	
Power		**0.85, 0.94**	**0.94, 0.97**	**0.98, 0.99**	**0.96, 0.99**	**0.94, 0.96**	** > 0.8**	256	PSD	5, 30 trials	
Power	(2,1)	–	–	**0.86, 0.83**	–	–	**0.85,0.80**	128	PSD	HC, ASD	[154]^
ERD/ERS	–	–	–	–	**0.90, 0.88** 0.66	–	–	64	PSD	Rest, MRBD, PMBR	[102]
	–	–	–	0.90*	–	–	–	58	PSD	topography	[103]^

A summary of test–retest reliability of some signal features is presented along with the signal processing method used to extract the feature and important notes about the experiment. Bold values denote good to excellent reliability

*HC* healthy controls, *ASD* autism spectrum disorder, *MRBD* movement related beta desynchronization, *PMBR* post movement beta rebound

*p = 0.001

^Records added manually

Spectral power features of EEG signals have been tested during rest [153, 154] and task (cognitive and motor) conditions for different cohorts of subjects [102, 103]. Some of the spectral features observed during these experiments are related to timed behavior of oscillatory activity due to cued experiments, such as event-related desynchronization of the Beta band (ERD and Beta rebound) [102] and topographical patterns of Alpha activity R = 0.9302, p < 0.001 [103].

Test–retest reliability for rest EEG functional connectivity has been explored for few of the estimators listed in section “Measures and methods based on neural activity using EEG/EMG”: (1) for a cohort of people with Alzheimer by means of the amplitude envelope correlation (AEC), phase lag index (PLI) and weighted phase lag index (wPLI) [155]; (2) in healthy subjects using iCoh and PLI [156]; and (3) in infants, by studying differences of inter-session PLI graph metrics such as path length, cluster coefficient, and network “small-worldness” [60]. Reliability for upper limb CMC has not yet been documented (at least to our knowledge). However, an experiment involving testing reliability of CMC for gait reports low CMC reliability in groups with different ages [61].

## Integrated metrics

EEG and EMG measurements could be combined with kinematic and kinetic measurements to provide additional information about the severity of impairment and decrease the number of false positives from individual measurements [21]. This could further be used to explain abnormal relationships between brain activation, muscle activation and movement kinematics, as well as provide insight about subject motor performance during therapy [15]. The availability of EEG and EMG measures can also enhance aspects of biofeedback given during tests or be used to complement other assessments to provide a more holistic picture of an individual’s neurological function.

It has been shown that combining EEG, EMG, and kinematic data using a multi-domain approach can produce correlations to traditional clinical assessments, a summary of some of the reviewed studies is presented in Table 10. Belfatto et al. have assessed people’s ROM for shoulder and elbow flexion, task time, and computed jerk to measure people’s smoothness, while the EMG was used to measure muscle synergies, and EEG detected ERD and a lateralization coefficient [21]. Comani et al. used task time, path length, normalized jerk, and speed to measure motor performance while observing ERD and ERS during motor training [22]. Pierella et al. gathered kinematic data from an upper-limb exoskeleton, which assessed the mean tangential velocity, path-length ratio, the number of speed peaks, spectral arc length, the amount of assistance, task time, and percentage of workspace, while observing EEG and EMG activity [18]. Mazzoleni et al. used the InMotion2 robot system to capture the movement accuracy, movement efficiency, mean speed, and the number of velocity peaks, while measuring brain activity with EEG [16]. However, further research is necessary to determine the effectiveness of the chosen metrics and methods compared to other more promising methods to assess function. Furthermore, greater consensus in literature is needed to support the clinical use of more reliable metrics. For example, newer algorithms to estimate smoothness such as spectral arc length have been shown to provide greater validity and reliability than the commonly used normalized jerk metric. Despite this evidence, normalized jerk remains a widely accepted measure of movement smoothness.

**Table 10:** Summary of multi-domain assessment studies

Biomechanical	Neurological signal features	Clinical test	Method combination	Result	Refs.
– Movement accuracy – Time of execution – Mean speed – Mean abs value of distance between trajectory – Number peaks speed – Spectral arc-length	*EEG:* – SVD topography – Coefficient of time frequency variation all bands *EMG:* – Muscle group activation – Spatial synergies NNMF*	– FMA longitudinally – Grip force test	– Correlation analysis – CCA – PCA	– All domains changed due to rehab – PCA metric combining information correlates significatively with FMA and grip force	[18]
– Range of motion (RoM) – Movement duration – Normalized Jerk	*EEG* – ERD latency, peak frequency, topography – BSI *EMG:* – Muscle activation – Spatial synergies NNMF*	Evolution of – FMA – WOLF Through therapy	– Domain to clinical test (did not combine information)	– All domains affected by rehab	[21]
– Range of motion (RoM) – Mean absolute of the distance (deviation from a straight line) – End-point error (aim) – Ratio of mean and peak speed – Mean-absolute jerk normalized by peak speed – Root mean square jerk normalized by duration of movement	*EEG:* – Rest power density – Relative and absolute Alpha power – Topography maps Alpha *EMG:* – Muscle group activation	– FMA – B&B – AS – Motricity index (MI) – Modified Barthel index (mBI)	– Domain to clinical test, did not combine information	– All domains affected by rehab	[122]
– Movement duration – Peak velocity – Index of curvature – Average jerk – Average inter-quartile range	*EEG:* – Alpha and Beta ERS/ERD features	– Relationship between kinematic data and neural data	– Correlation analysis	Significant correlation: – Peak Velocity & Alpha ERD – Index of curvature & Beta ERS – Index of curvature & Beta ERD – Average jerk & Alpha ERS – Average jerk & Alpha ERD – Inter-quartile range_x_ Beta ERS – Inter-quartile range_z_ Beta ERS – Inter-quartile range_x_ Beta ERS-ERD – Inter-quartile range_z_ Beta ERS-ERD	[15]
– Movement duration – Path length – Normalized Jerk Speed	*EEG* topography – Alpha and Beta ERS/ERD – BSI	– NHPT – Motricity index (mi) – Barthel Index – FIM – Canadian stroke scale	– Separate analysis for each domain	– All domains affected by rehab	[22]
– Movement accuracy – Movement efficiency – Mean speed – Number of velocity peaks	*EEG:* – ERD % – Motor potential amplitude	– Motor status score – MAS – Range of motion	– Correlation analysis	– Movement efficiency and number of velocity peaks quantify movement execution for kinematic – The motor potential amplitude and ERD% are significant parameters	[16]

Information about metrics from each domain and about how information is combined

*NNMF* non-negative matrix factorization algorithm

## Discussions and conclusions

In this paper we reviewed studies that used different sensor-acquired biomechanical and electrophysiological signals to derive metrics related to neuromuscular impairment for stroke survivors; such metrics are of interest for robotic therapy and assessment applications. To assess the ability of a given measure to relate with impairment or motor outcome, we looked for metrics where results have been demonstrated to correlate or predict scores from established clinical assessment metrics for impairment and function (validity). Knowing that a metric has some relationship with impairment and function (i.e., that it is valid) is not enough for it to be used in clinical settings if those results are not repeatable (reliable). Thus, we also reviewed the reliability of metrics and related signal features looking for metrics which produce similar results for the same subject during different test sessions and for different raters. With this information, researchers can aim to use metrics that not only seem to be related with stroke, but also can be trusted, with less bias, and with a simpler interpretation. The main conclusions of this review paper are presented as answers to the following research questions.

### Which biomechanical-based metrics show promise for valid assessment of function and impairment?

Metrics derived from kinematic (e.g., position & velocity) and kinetic (e.g., force & torque) sensors affixed to robotic and passive mechanical devices have successfully been used to measure biomechanical aspects of upper-extremity function and impairment in people after stroke. The five common metrics included in the reviewed studies measured the number of velocity peaks (~ 9 studies), path-length ratio (~ 8 studies), the maximum speed of the arm (~ 7 studies), active range of motion (~ 7 studies), and movement time (~ 7 studies). The metrics are often compared to an established clinical assessment to determine validity of the metric. According to the review study by Murphy and Häger, the Fugl-Meyer Assessment for Upper Extremity had significant correlation with movement time, movement smoothness, peak velocity, elbow extension, and shoulder flexion [66]. The movement time and smoothness showed strong correlation with the Action Research Arm Test, whereas speed, path-length ratio, and end-point error showed moderate correlation. Tran et al. reviewed specifically validation of robotic metrics with clinical assessments [57]. The review found mean speed, number of peak velocities, movement accuracy, and movement duration to be most promising metrics based on validation with clinical assessments. However, the review mentioned that some studies seem to conflict on the correlation between the robotic metric and clinical measures, which could be due to assessment task, subject characteristics, type of intervention, and robotic device. For further information about the validation of sensor-based metrics, please refer to the previously mentioned literature reviews [57, 66].

### Which biomechanical-based metrics show promise for repeatable assessment?

Repeatable measures, in which measurement taken by a single instrument and/or person produce low variation within a single task, are a critical requirement for assessment of impairment and function. The biomechanical based metrics that show the most promise for repeatability are range of motion, mean speed, mean distance, normal path length, spectral arc length, number of peaks, and task time. Two or more studies used these metrics and demonstrated good and excellent reliability, which implies the metric is robust against measurement noise and/or disturbances. Since the metrics have been used on different measuring instruments, the sensors’ resolution and signal-to-noise ratio appear to have a minimal impact on the reliability. However, more investigation is needed to confirm this robustness. In lieu of more evidence, it is recommended that investigators choose sensors similar or superior in quality to those used in the measuring devices presented in Tables 7 and 8 to achieve the same level of reliability.

### What aspects of biomechanical-based metrics lack evidence or require more investigation?

Although many metrics (see previous section) demonstrate good or excellent repeatability across multiple studies, the evidence for reproducibility is limited to single studies. When developing a novel device capable of robotic assistance and assessment, researchers have typically focused their efforts to create a device capable of repeatable and reliable measurements. However, since the person administering the test is using the device to measure the subject’s performance, the reproducibility of the metric must also be considered. The reproducibility of a metric is affected by the ease-of-use of the device; if the device is too complicated to setup and use, there is an increased probability that different operators will observe different measurements. Also, the operator’s instructions to the subject affects the reproducibility, especially in the initial sessions, which may lead to different learning effects, and different assessment results. More studies are needed across multiple sites and operators to determine the reproducibility of the biomechanical metrics reviewed in this paper.

### Which neural activity-based metrics (EEG & EMG) show the most promise for reliable assessment?

Electrical neurological signals such as EEG and EMG have successfully been used to understand changes in motor performance and outcome variability across all stages of post-stroke recovery including the first few hours after onset. Experimental results have shown that metrics derived from slow frequency power (delta power, relative delta power, and theta power), and power ratio between slow and fast EEG frequency bands like *DAR* and *DTABR* convey useful information both about current and future motor capabilities, as presented in Table 4 and Table 11 in Appendix 1. Multimodal studies using robotic tools for assessment of motor performance have expanded the study of power signal features in people who suffered a stroke in the chronic recovery stage by studying not only rest EEG activity but also task-related activity [19, 21, 122]; *ERD-ERS* features like amplitude and latency along with biomechanical measures have been shown to correlate with clinical measures of motor performance and to predict a person’s response to movement therapies. EEG power features in general have been found to have good to excellent reliability for test–retest conditions among different populations, across all frequency bands of interest (see Table 9).

Functional connectivity (i.e., non-directed connectivity) expands the investigative capacity of EEG measurements, enabling analyzing the brain as a network system by investigating the interactions between regions of interest in the brain while resting or during movement tasks. Inter-hemispheric interactions (interactions between the same ROI in both hemispheres) and global interactions (interactions between the entire brain and an ROI) reported as power or graph indices in Beta and Gamma bands have fruitfully been used to explain motor outcome scores. Although results seem promising, connectivity reliability is still debated with results ranging mostly between moderate to good reliability only for a few connectivity estimators (*PLI,* *wPLI* and *iCoh*).

### Which neural activity-based metrics (EEG and EMG) lack evidence or require more investigation?

EEG and EMG provide useful non-invasive insight into the human neuromuscular system allowing researchers to make conjectures about its function and structure; however, interpretation of results based on these measures solely must be carefully analyzed within the frame of experimental conditions. Overall, the field needs more studies involving cohorts of stroke survivors to determine the reliability (test–retest) of metrics derived from EEG and EMG signal features that have already shown validity in academic studies.

Metrics calculated from power imbalance between interhemispheric activity like *BSI*, *pwBSI* and *PRI* [62, 73, 124] are a great premise to measure how the brain relies on foreign regions to accomplish tasks related with affected areas. A battery of diverse estimators for connectivity, especially those of effective (directed) connectivity, open the door to investigations into the relationship between abnormal communication of regions of interest and impairment (see Table 5 and Table 12 in Appendix 2). These metrics, although valid have yet to be tested in terms of reliability in clinical use. Reliability for connectivity metrics should specify which estimator was used to derive the metric.

CMC is another exciting neural-activity-based metric lacking sufficient evidence to support its significance. CMC considers and bridges two of the most affected domains for motor execution in neuromuscular system, making it a good candidate for robotic-based therapy and assessment of survivors of stroke [147]. Although features in the Beta and Gamma bands seem to be related to motor impairment, there is still not agreement about which one is most closely related to motor outcomes. Studies reviewed in this paper considered cortical spatial patterns of maximum coherence, peak frequency shift when compared to healthy controls, latency for peak coherence, among others (see Table 6 and Table 13 in Appendix 3). However, when comparing to motor outcomes, results are not always significant, and test–retest reliability for this metric is yet (to our knowledge) to be documented for the upper extremity (see [61] for a lower-extremity study).

### What standards should be adopted for reporting biomechanical and neural activity-based metrics and their reliability?

For metrics to be accepted as reliable in the clinical field, researchers are asked to follow the guidelines presented in Koo and Li [68], which provide guidance on which ICC model to use depending on the type of reliability study and what should be reported (e.g., the software they used to compute the ICC and confidence interval). In the papers reviewed, some investigated the learning effects of the assessment task and checked for consistency rather than agreement (see Table 7). However, the learning effects should be minimal in a clinical setting between each session, and potential effects should be taken into consideration during protocol design; common practices to minimize the implications of learning effects is to allow practice runs by the patients [99, 122] and to remove the first experimental runs [81, 85]. By removing this information, signal analysis focuses performance of learned tasks with similar associated behaviors. Therefore, to demonstrate test–retest reliability (i.e., repeatability), the researcher should be checking for absolute agreement. Also, as can be seen in Tables 7 and 8, there does not seem to be a standard on reporting ICC values. Some researchers report the confidence interval of the ICC value, while others do not. It was also difficult to determine the ICC model used in some of the studies. Therefore, a standard on reporting ICC values is needed to help readers understand the ICC used and prevent bias (see [68] for suggestive guideline on how to report ICC scores). Also, authors are asked to include the means of each individual session or rater would provide additional information on the variation of the means between the groups. The variation between groups can be shown with Bland–Altman plot, but readers are unable to perform other forms of analysis. To help with this, data from studies should be made publicly available to allow results to be verified and enable further analysis in the future.

### When is it advantageous to combine biomechanical and neural activity-based metrics for assessment?

Biomechanical and neural activity provide distinct but complementary information about the neuro-musculoskeletal system, potentially offering a more complete picture of impairment and function after stroke. Metrics derived from kinematic/kinetic information assess motor performance based on motor execution; however, compensatory strategies related to stroke may mask underlying neural deficits (i.e., muscle synergies line up to complete a given task) [18, 21, 69–72, 122]. Information relevant to these compensatory strategies can be obtained when analyzing electrophysiological activity, as has been done using connectivity [59, 107], CMC [147, 148] and brain cortical power [91].

Combining signals from multiple domains, although beneficial in the sense that it would allow a deeper understanding of a subject’s motor ability, is still a subject of exploration. Experimental paradigms play an important role that influences the decision of feature selection; increasing the dimensionality of signals may provide more useful information for analysis, but comes at the expense of experimental costs (e.g., hardware) and time (e.g., subject setup). With all this in mind, merging information from different domains in the hierarchy of the neuro-musculoskeletal system may provide a more comprehensive quantitative profile of a person’s impairment and performance. Examples of robotic multidomain methods such as the ones in [18, 21], highlight the importance of this type of assessment for monitoring and understanding the impact of rehabilitation in chronic stroke survivors. In both cases, these methodologies allowed pairing of observed behavioral changes in task execution (i.e., biomechanical data) with corresponding functional recovery, instead of adopted compensation strategies.

### What should be the focus of future investigations of biomechanical and/or neural activity-based metrics?

Determining the reliability and validity of sensor-based metrics requires carefully designed experiments. In future investigations, experiments should be conducted that calculate multiple metrics from multiple sensors and device combinations, allowing the effect of sensor type and quality on the measure’s reliability to be quantified. After the conclusion of such experiments, researchers are strongly encouraged to make their anonymized raw data public to allow other researchers to compute different ICCs. Performing comparison studies on the reliability of metrics will produce reliability data to expand Tables 7, 8, 9 and improve our ability to compare similar sensor-based metrics. Additional reliability studies should also be performed that include neural features of survivors of stroke, with increased focus on modeling the interactions between these domains (biomechanical and neural activity). It is also important to understand how to successfully combine data from multimodal experiments; many of the studies reviewed in this paper recorded multidimensional data, but performed analysis for each domain separately.

## Data Availability

Not applicable.

## Appendix 1

See Table 11.

**Table 11:** Extended version of EEG signal power metrics and their relationship to motor function or outcome in stroke

Correlation with clinical measures	EEG metric	Severity	Stage	Age mean (std), [range]	Stroke type	M/F	# Sub	Year	Author	Ref.
First noticeable electrical changes during stroke	Global alpha and beta power	NIHSS: 14(6.3)	Acute	69.3(9.9)	IS	11/7	18	2016	Finnigan	[58]
105-day FIM FA	Global alpha and beta power	NIHSS: 9(7.7)	Acute	71(14)	IS	13/12	25	2014	Schleiger	[53]
12-months NIHS	Global alpha and beta power	NIHSS: 14(9)	Acute	67.4(11.9)	IS	38/112	151	2018	Bentes	[96]
3-months mR	Delta power, Theta power	mRS: 2.7(1.1)	Acute	65(10)	IS	11/17	28	2007	Cuspineda	[36]
Delayed Cerebal Ischemia (DCI)	Delta power, Theta power	NA	Acute	55[18 82]	IS	26/12	34	2004	Claassen	[43]
1-month NIHS	Delta power, Theta power	NIHSS: 16(9)	Acute	74.5[55 87]	IS	6/5	11	2004	Finnigan	[52]
30 and 90 days mBI and mRS	Delta power, Theta power	NIHSS: 6.5(6.4)	Acute	65.7(17)	IS(12) and HS(4)	7/9	16	2020	Rogers	[121]
6-months mR	DTR, DAR, DTABR	NA	Acute	75[24.5 89]	IS	55/55	110	2011	Sheorajpanday	[105]
90-days MoC	DTR, DAR, DTABR	NIHSS: 5.7(6.2), mRS: 1.9(1.4)	Acute	65.95(15.78)	IS(15) and HS (4)	12/7	19	2017	Aminov	[54]
NIHSS evolution 3 weeks to 6-mont	DTR, DAR, DTABR	NIHSS: 5 [3.5 7.5]	Acute- Subacute	67(11)	IS	17/24	41	2020	Saes	[120]
2-months FMUE	BSI, pdBSI	NIHSS: 13	Chronic	61 [37 81]	IS	9/11	20	2013	Dubovik	[104]
6-months mR	BSI, pdBSI	NIHSS: 2.4(2.5), FMUE: 43(22)	Chronic	60.6 [48 77]	IS	6/15	21	2019	Saes	[125]
WMFT and FM	Global alpha and beta power	mild to moderate paresis	Chronic	59.5(13)	NA	3/1	14	2018	Bertolucci	[47]
shape and delay of ERD-ERS	Global alpha and beta power	barthel: 11, FIM: 60	sub acute	75	NA	0/1	1	2015	Comani	[101]
motor therapy gain	Global delta	Chronic: 8, subacute: 29	Chronic and sub acute	56 and 75	IS	1/1	2	2015	Sale P	[122]
theta band BSI predictors 6-months FM-UE	BSI	FMUE: 21, ARAT: 3.5	Acute, Subacute	67.3(11.4)	IS	16/23	39	2021	Saes	[124]
FMUE change	DAR, DTABR	FMUE: 42(17)	Chronic	55.7(16)	IS (8) and HS (2)	2/8	10	2017	Trujillo	[99]

*IS* ischemic stroke, *HS* hemorrhagic stroke, *M* male, *F* female

## Appendix 2

See Table 12.

**Table 12:** Extended version of metrics from EEG brain connectivity and main findings related to motor function

Correlation with clinical measures	EEG metric	Severity	Stage	Age mean (std), [range]	Stroke type	M/F	# Sub	Year	Author	Ref.
Significant differences between people with stroke and control	Delta and Alpha2 small-worldness	NIHSS: 10.2(7)	Acute	66.3(10.6)	IS	7/23	30	2017	Caliandro	[108]
Predictor of FM-UE score	Maximum coherence PLI alpha band	FMUE: 39.4(10.3)	Sub-Acute	64.2(12.4)	NA	3/7	10	2020	Riahi	[139]
Motor therapy gains, FMA predictor	Ipsilesional High beta M1-PM and network to M1 coherence	FMUE: [22 55]	Chronic	54 (16)	NA	6/6	12	2015	Wu	[94]
FM-UE and hand recovery	Interhemispheric M1 Beta PDC	FMUE: 31(5.6)	Chronic	60(13)	IS	1/2	3	2018	Eldeeb	[109]
ARAT and FM-UE	Interhemispheric M1 Beta dwPLI	FMUE: 30.3(12.5), ARAT: 18.7(19)	Chronic	64.4 (11.1)	IS and HS	10/26	36	2020	Hordacre	[138]
FMA-UE, Nine Hole Peg Test	Motor cortex Weighted node Degree (WND) from iCoh beta band	NIHSS: 13[3 27]	Chronic	60.7 [37 81]	IS	9/15	24	2015	Pietro	[38]
3-months FMA	Global alpha connectivity with motor cortex	NIHSS: 13	Chronic	61 [37 81]	IS	9/11	20	2013	Dubovik	[104]
Cortico Spinal Tract (CST) integrity, impairment	Beta, Gamma Normalized inter hemispheric strength (nIHS) from PDC	FMUE: 15(12)	Sub acute	66 (8)	NA	NA	30	2019	Pichiorri	[136]
Survivors of stroke have less-efficient networks vs. unimpaired	Global and local Beta/Gamma bands network efficiency	NA	Chronic	65	NA	0/1	1	2009	Fallani	[106]
FM-UE	Global and local Beta/Gamma bands network efficiency	FMUE: 23.6(10)	Chronic	57.90(12.44)	IS(24) and HS(6)	9/21	30	2017	Phillips	[135]

*IS* ischemic stroke, *HS* hemorrhagic stroke, *M* male, *F* female

## Appendix 3

See Table 13.

**Table 13:** Extended version of metrics from EEG-EMG coherence (CMC) and main findings related to motor function

Correlation with clinical measures	EEG metric	Severity	Stage	Age mean (std), [range]	Stroke type	M/F	# Sub	Year	Author	Ref.
Flexion synergy	Beta, Gamma CMC	MMSE: 28(1)*	Chronic	54.5(6.9)	NA	1/11	12	2018	Pan	[93]
FMUE	Beta, Gamma CMC	FMUE: 16.2(6.8)	Chronic	57(11)	IS(3) and HS(5)	1/7	8	2017	Belardinelli	[37]
Function recovery, FMA-UE	Beta CMC	FMUE: 15	Subacute	35	NA	0/1	1	2018	Zheng	[141]
Compensation of healthy limb	Gamma interhemispheric disparity	ARAT: [10 30]	Chronic	52(8.1)	NA	5/6	11	2018	Bao	[145]
Characterization of CMC from stage to stage	CMC frequency peak	MMSE: 27(2)*	Acute to chronic	71(11)	IS	6/6	11	2014	Von Carlowitz-Ghori	[146]
Non-significant differences among stages	CMC frequency peak	mild to moderate hand weakness	Chronic	59(15)	IS	2/13	15	2017	Larsen	[112]
Co-contraction	CMC frequency peak	FMUE: 33.6(10), MAS: 1.3(0.8),1.4(0.7),1.6(0.6)**	Chronic	56.5(9.5)	IS(7) and HS (7)	3/11	14	2020	Guo	[149]
FMUE	CMC topographical patterns	FMUE: 55	Acute to chronic	58.3 [52 64]	IS	NA	11	2016	Krauth	[144]
Level of impairment	CMC topographical patterns	ARAT: 45(19)	Subacute	52(14)	NA	6/19	25	2013	Rossiter	[142]

*IS* ischemic stroke, *HS* hemorrhagic stroke, *M* male, *F* female, * MMSE: mini mental state examination score, ** MAS finger, wrist, elbow

## Abbreviations

ADL: Activities of daily living
AEC: Amplitude envelope correlation
ARAT: Action research arm test
aROM: Active range of motion
ASD: Autism spectrum disorder
B&B: Box and Blocks test
BSI: Brain Symmetry Index
CCA: Canonical correlation analysis
CMC: Corticomuscular coherence
CST: Cortico-spinal tract
DAR: Delta-alpha ratio
DCI: Delayed cerebral ischemia
dDTF: Direct directed transfer function
DOF: Degree of freedom
DTABR: (Delta + Theta)/(Alpha + Beta)
DTF: Directed transfer function
DTR: Delta-theta ratio
EEG: Electroencephalography
EMG: Electromyography
ERD: Event related desynchronization
ERS: Event related synchronization
ffDTF: Full frequency directed transfer function
FIM-FAM: Functional independence measure and functional assessment measure
FMA or FMA-UE: Fugl-Meyer assessment for upper extremity
GMA: Generalized Measure of Association
GPDC: Generalized partial directed coherence
ICC: Intra-class correlations
iCoh: Imaginary part of coherence
M1: Primary motor cortex
MA: Modified Ashworth
mBI: Modified Barthel Index
mCoh: Multiple coherence
MI: Motricity Index
MoCa: Montreal Cognitive Assessment
MRBD: Movement related beta desynchronization
MRI: Magnetic resonance imaging
mRS: Modified Ranking Scale
nIHS: Normalized interhemispheric strength
NIHSS: National Institutes of Health Stroke Scale
NNMF: Non-negative matrix factorization algorithm
PCA: Principal component analysis
pCoh: Partial coherence
PDC: Partial directed coherence
PLI, wPLI, dwPLI: Phase lag index, weight phase lag index, debiased weighted phase lag index
PM: Premotor area
PMBR: Post movement beta rebound
PRI: Power Ratio Index
pROM: Passive range of motion
qEEG: Quantitative EEG
rCBF: Regional cerebral blood flow
ROI: Region of interest
rPDC: Renormalized partial directed coherence
SVD: Singular value decomposition
WMFT: Wolf motor function
WND: Weighted Node Degree Index
